# A Path-Planning Method to Significantly Reduce Local Oscillation of Manipulators Based on Velocity Potential Field

**DOI:** 10.3390/s23239617

**Published:** 2023-12-04

**Authors:** Xianlun Wang, Qingsong Wu, Tianyu Wang, Yuxia Cui

**Affiliations:** 1 College of Electromechanical Engineering, Qingdao University of Science and Technology, Qingdao 266000, China; xlwang@126.com (X.W.); 2021030025@mails.qust.edu.cn (Q.W.); tywang124@163.com (T.W.); 2 Collaborative Innovation Center for Intelligent Green Manufacturing Technology and Equipment of Shandong, Qingdao 266000, China

**Keywords:** human-robot collaboration, velocity potential field method, real-time obstacle avoidance, trajectory planning

## Abstract

The robotics industry and associated technology applications are a vital support for modern production and manufacturing. With the intelligent development of the manufacturing industry, the application of collaboration robots and human-robot collaboration technology is becoming more and more extensive. In a human-robot collaboration scenario, there are uncertainties such as dynamic impediments, especially in the human upper limb, which puts forward a higher assessment of the manipulator’s route planning technology. As one of the primary branches of the artificial potential field (APF), the velocity potential field (VPF) offers the advantages of good real-time performance and convenient mathematical expression. However, the traditional VPF algorithm is prone to local oscillation phenomena near obstacles, which degrades the smoothness of the movement of the manipulators. An improved velocity potential field algorithm is proposed in this paper. This method solves the problem of sudden velocity change when the manipulator enters and departs the region of the potential field by setting new functions for attraction velocity and repulsion velocity functions. A virtual target point construction method is given to overcome the local oscillation problem of the manipulators near obstacles. The simulation and practical findings of the manipulators reveal that the improved VPF algorithm can not only avoid collision but also effectively reduce the local oscillation problem when dealing with the human upper limb as a dynamic obstacle. The implementation of this algorithm can increase the safety and real-time performance of the human-robot collaboration process and ensure that the collaborative robot is safer and smoother in the working process.

## 1. Introduction

Industrial robots are becoming more and more prevalent. The interaction between robots and humans has gone through four stages: competition, coexistence, collaboration, and co-working [1]. A new generation of collaborative robots with related application technologies has emerged to adapt to the manufacturing and service industries’ changing trends. These robots facilitate a new stage of collaboration between humans and robots [2,3,4]. Currently, businesses are increasingly showing interest in using flexible production and collaborative robots to improve their manufacturing processes. Collaborative robots, particularly lightweight and flexible ones, boost work efficiency by complementing human labor with machine precision and capacity [5,6]. Human-robot collaboration requires critical research in robot motion planning. Robotics motion planning is a highly active research area in the field of human-robot collaboration [7]. Real-time robot path planning is vital to ensure that robots can efficiently and safely avoid obstacles and reach their target destinations. This can be achieved either by maintaining the continuity of velocity and acceleration or by minimizing the trajectory re-planning. These algorithms often face issues such as intricate calculations and unstable search paths. Currently, most path-planning algorithms operate in static low-dimensional environments. Fewer algorithms are suitable for high-dimensional environments due to their limitations, such as complex calculations or unstable search paths [8,9]. Consequently, it is crucial to conduct research into real-time robot path planning to ensure safe navigation and obstacle avoidance, while maintaining both smooth and continuous velocity and acceleration and minimizing the need for trajectory re-planning to enhance planning efficiency [10,11,12].

Robot path planning is a crucial research domain in robotics, as robots can perform a plethora of tasks efficiently, via both offline teaching and online autonomous planning, without depending on path-planning technologies. However, as robots move towards collaborating with humans, including in domestic scenarios, the necessity for local path planning is accentuated. The necessity for path planning has, therefore, become a vital research area in robotics. Common path-planning algorithms include A* [13,14], PRM [15,16], fast extended random tree (RRT) [17,18], and APF [19,20,21]. APF guides the manipulator to reach the target or circumvent obstacles through virtual potential fields, force, or velocity potential fields. The artificial potential field (APF) algorithm is a classical method for robot trajectory planning, which is characterized by good real-time performance and minimal computational consumption. Hence, numerous academics have improved the APF algorithm. To accomplish joint-limit and obstacle avoidance, Ye et al. [22] integrated the quadratic programming-based control strategy with the APF approach. Yang et al. [23] modified the action range of the gravitational field and the direction of the original algorithm. When the UAV is caught in a local minimum, a rectangular model centered around the initial target and the corner point is selected as a temporary target based on the evaluation function, thereby the local minima problem is solved. The artificial velocity potential field approach was utilized by Christoff et al. [24] to execute the movements and trajectory planning of two 6-DoF surgical manipulators. Min et al. [25] proposed an experience-based modified potential field method that uses previous experience to achieve obstacle avoidance when the type of obstacle is identified as a known type, or using the modified artificial potential field method otherwise.

As one of the branches of APF, the VPF offers the advantages of a simple potential field, more efficiency, and easier computation. In the manipulator’s path planning, the VPF is more frequently utilized. It is worth noting that the elastic band methods which are based on curve shortening flows in a potential field also adopt the idea of the potential field method. However, compared to the VPF method, the VPF has a simpler algorithmic function construction method and higher algorithmic efficiency. However, the VPF still suffers from the problems of quickly falling into local minima and oscillating in the presence of obstacles. Hence, numerous academics have improved the VPF algorithm. Xu et al. [26] established a saturation function in the attraction velocity potential field function and designed a spring-damping mechanism in the repulsion velocity potential field. This strategy makes the obstacle-avoidance procedure smoother and eliminates the vibration issue around the obstacles. Zhang et al. [27] presented a method to apply the velocity potential field method to the obstacle avoidance strategy of a dual-arm robot. The attraction and repulsion functions are mapped onto the joint space, and a difference function is employed to address the collision avoidance problem between two manipulators in the context of the dual-arm robot. The modified VPF algorithm tackles the drawbacks of being prone to local minima and lack of relevance in confronting dynamic impediments. The VPF can also be utilized to achieve stable target tracking for spray robots. Xia et al. [19] used the VPF to ensure that the target loading of spray robots is at the same relative velocity as the target, which enhances the robustness of the target positioning and tracking control of the spray robot. Zhao et al. [28] used the VPF to ensure that the spray robot’s target loading is at the same relative velocity as the target, boosting the robustness of the spray robot’s targeting and tracking control. Ouyang et al. [29] incorporated virtual velocity vectors into the classic VPF method for the manipulator’s path planning in industrial contexts. The efficiency of trajectory generation may be enhanced by employing this method, while the joint space velocity and the right-angle coordinate space velocity are continuously smooth, which meets the requirements for application in industrial settings.

Numerous researchers have improved the VPF; however, current methods do not deal with the dynamic obstacle-avoidance problem of manipulators in human-robot collaboration well. These methods cannot match the real-time and safety criteria for manipulator obstacle avoidance in robot collaboration scenarios. Hence, in this research, an improved VPF algorithm is proposed for the dynamic obstacle-avoidance problem of manipulators in human-robot collaboration settings. This method eliminates the local oscillation problem in the standard VPF method and makes the path smoother. It addresses the safety and real-time criteria of dynamic obstacle avoidance in human-robot collaboration situations, thereby increasing the efficiency of human-robot cooperation. The rest of this paper is structured as follows. In Section 2, a path-planning algorithm for a manipulator in 3D space based on the velocity potential field is proposed. The performance of the proposed algorithm and the results will be analyzed in Section 3. The whole discussion is summarized, and a conclusion is presented in Section 4.

## 2. Improved Velocity Potential Field for Local Oscillation

The current path-planning algorithm utilized for autonomous obstacle-avoidance research in static obstacle situations is suited more towards two-dimensional objects, such as unmanned aerial vehicles, unmanned vehicles, and mobile robots. As space dimensions increase, this algorithm becomes increasingly complex, and it becomes difficult to ensure real-time performance. To overcome these constraints, this paper proposes a path- planning algorithm for manipulators in dynamic environments founded on the principles of potential field methods. This algorithm guarantees the safety of the manipulator’s driving path while allowing the speed to be well-controlled or minimizing the speed re-planning process to ensure efficient operations.

### 2.1. Implementation of the Velocity Potential Field Method on the Manipulator

The core ideal of path planning using the velocity potential field is to convert the distance between the manipulator, target, or obstacle into a spatial velocity in Cartesian coordinates. This spatial velocity is then mapped to the robot’s joint space to obtain the joint velocity required to control the robot. This process is accomplished using the Jacobi matrix.

**Figure 1:**
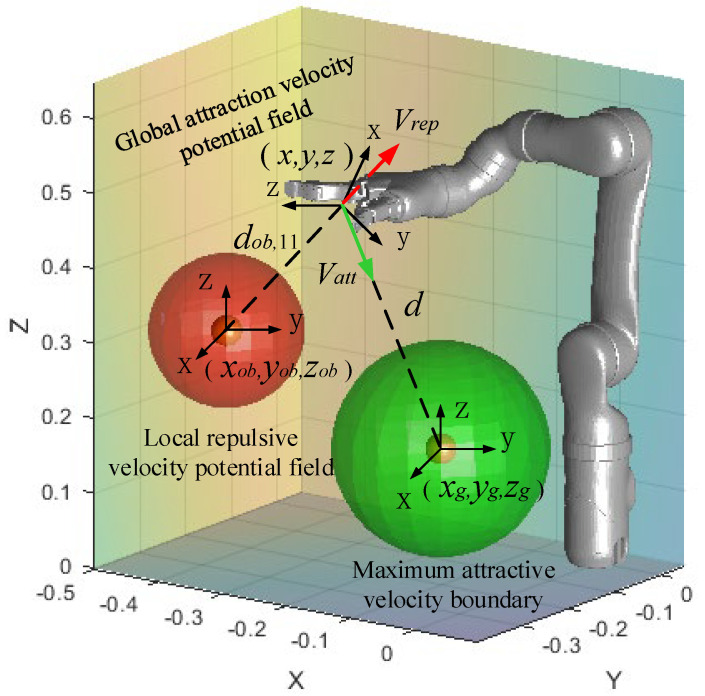
Obstacle-avoidance velocity generation of Mico2 manipulator.

The spatial velocity transformation for a manipulator typically requires a Jacobi non-square form, which consequently needs a solution via a non-square format Jacobi matrix [26]. Currently, the most popular method used in practical applications to obtain the pseudo-inverse solution is the Singular Value Decomposition (SVD) algorithm, which is used in this paper. This paper adopts an attractive velocity potential field as the global potential field, which defines the maximum attractive velocity boundary around the target object. Additionally, a local repulsive velocity potential field is established, centered around the obstacles. The manipulator in space is influenced by the attractive velocity $V_{att}$ and moves towards the target object. If the manipulator enters the range of the local potential field, it is influenced by the repulsive velocity $V_{rep}$ and moves away from the obstacles. Finally, the overall path planning is completed. The overall potential field model is depicted in Figure 1.

### 2.2. Improved Velocity Potential Field

The attraction velocity potential field function is defined as a vector that extends from the end point of the robot to the target, and points in the direction of attraction velocity. The magnitude of the attraction velocity is determined by the distance between the target and the end of the manipulator. The direction of the attraction velocity remains from the end of the manipulator pointing to the target, with a size that follows the principle of “Slow-Fast-Slow”. The magnitudes of the velocity components in the *x*, *y* and *z* directions are determined as the respective percentages of $\lambda_{\left(x,y,z\right)}$. The result is shown in Equation (1).

(1) $$\lambda_{\left(x,y,z\right)}=\frac{\left|v_{att}\right|}{\left|v_{attx}\right|+\left|v_{atty}\right|+\left|v_{attz}\right|}$$

The distance separating the end of the manipulator from the target point determines three distinct zones: acceleration section $\left[d_{0},d_{max}\right]$, uniform speed section $\left[d_{max},d_{1}\right]$, and deceleration section $\left[d_{1},0\right]$. For the *x*-direction, the attraction velocity function can be defined as:

(2) $$V_{att}=\left\{\begin{array}{l} sign(\nu_{attx})\cdot \alpha \cdot \lambda_{x}\cdot d_{max}\cdot sin(\frac{\pi (d_{0}-d)}{2(d_{0}-d_{max})}),d\in [d_{0},d_{max}]\\ sign(\nu_{attx})\cdot \alpha \cdot \lambda_{x}\cdot d_{*max},d\in [d_{*max},d_{1}]\\ sign(\nu_{atx})\cdot \alpha \cdot \lambda_{x}\cdot d_{max}\cdot sin(\frac{\pi d}{2d_{1}}),d\in [d_{1},0] \end{array}\right.$$

In the *x*-direction, $sign(v_{attx})$ represents the positive and negative values of velocity, α represents the attraction velocity intensity factor, and $d_{max}$ represents the maximum attraction velocity boundary radius. The product of α and $d_{max}$ is significant as it defines the maximum velocity the robot can achieve.

By introducing trigonometric functions, the manipulator can accelerate the attraction speed from zero to the maximum speed allowed while moving away from the target point. Once the maximum speed is achieved, the manipulator moves at a uniform speed for a specified distance.

**Figure 2:**
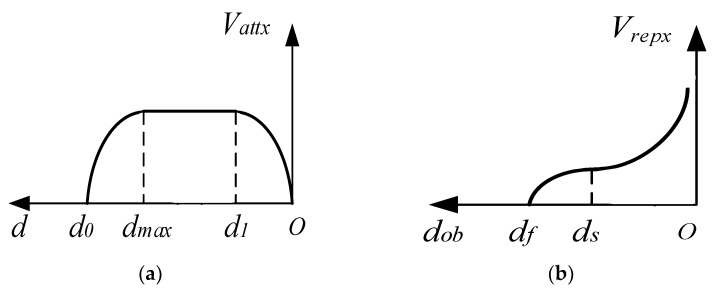
Attraction and repulsive velocity curve model of x direction. (**a**) Attraction velocity; (**b**) Repulsive velocity.

**Figure 3:**
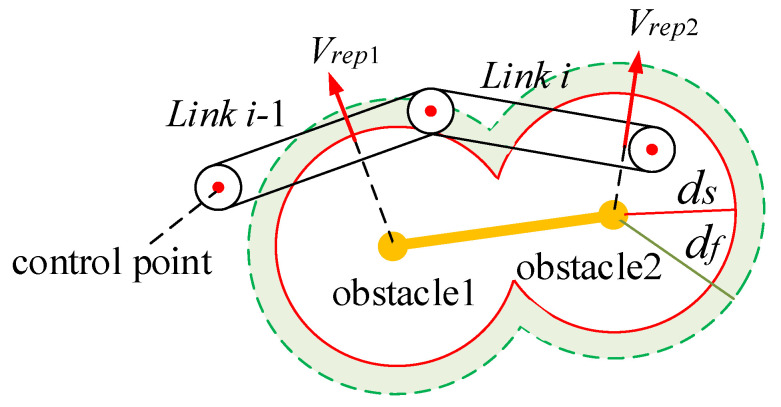
Multi-obstacle repulsive velocity potential field model.

As the manipulator approaches the target point, the attraction speed gradually decreases to zero. This S-curve-like velocity design scheme adjusts its velocity amplitude to approach the target in almost a straight line when there are no obstacles, avoiding the problem of oversized initial velocity or limited velocity, which fails to solve the problem of sudden change in initial speed while ensuring the mission’s efficiency. Figure 2a shows the Cartesian velocity profile model in the x-direction. During the operation of a manipulator, there is a situation in which a repulsive speed potential field is simultaneously applied to the manipulator by multiple obstacles, as shown in Figure 3. When multiple obstacle points generate repulsion speed potentials for the manipulator, the priority obstacle avoidance lies in selecting the speed of the joint closest to the obstacle, as outlined in Figure 3. Abrupt entry or exit from the repulsive velocity potential field will cause a sudden change in the total velocity imparted on the manipulator, which may cause an impact. The repulsive velocity potential field is partitioned around each obstacle. The repulsion zone is within the potential field with $d_{s}$ representing its radius, while the buffer zone outside the potential field is established with $d_{f}$ as the radius. The buffer velocity function alters the repulsion velocity close to zero when the control point enters the buffer zone but remains outside the repulsion zone, minimizing the effects on the manipulator caused by sudden velocity changes. The entrance into the repulsion zone is subjected to normal repulsive velocity.

The repulsive velocity function for the *x*-direction can be defined as:

(3) $$V_{repx}\left\{\begin{array}{l} 0,d_{ob}>d_{f}\\ \beta \cdot sin[(1-\frac{d_{ob}-d_{s}}{d_{f}-d_{s}}).\frac{\pi }{2}].\frac{\nu_{repx}}{d_{ob}^{2}},d_{s}<d_{ob}\leq d_{f}\\ \beta \cdot (\nu_{repx}/d_{ob}^{2}),d_{ob\leq }d_{s} \end{array}\right.$$

The distance between the obstacle and the closest control point of the manipulator is denoted by $d_{ob}$, while β represents the intensity factor of the repulsive velocity. The repulsive velocity model is depicted in Figure 2b. After being converted through the Jacobi matrix, the attraction and repulsion velocities are used to obtain the velocities in the joint space, as shown below:

(4) $$\dot{\theta }=piv(J_{i}^{-1})\cdot V_{rep(x,y,z)}+piv(J_{6}^{-1})\cdot V_{at(x,y,z)}$$

### 2.3. Local Oscillation and Virtual Target Point

**Figure 4:**
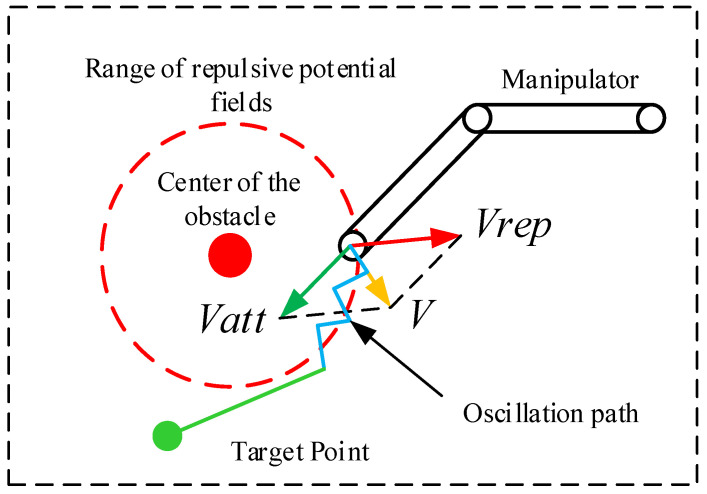
Motion path of the manipulator during local oscillation.

Since the artificial potential field method is based on the idea of the potential field method, whether the force potential field method or the velocity potential field method is utilized, the direction of the force control or velocity control of the manipulator is unidirectional and abrupt. Therefore, the speed of the manipulator will oscillate with the appearance and disappearance of the repulsive speed. When the manipulator experiences repulsion from an obstacle in a direction opposite to the attraction of a target object, or when the angle between the two directions is excessively large, it can result in the manipulator repeatedly leaving the repulsive velocity potential field range during obstacle avoidance and re-entering due to the attraction, causing local oscillation, as depicted in Figure 4. The virtual target-point-based processing technique is one of the key strategies to cope with the local oscillation problem created by the artificial potential field method. Other processing strategies have certain inherent issues. For example, the computation is more complicated, the real-time performance is not acceptable, and it generates a huge instantaneous force or velocity on the robotic arm, which compromises the safety of the operation of the robotic arm. Therefore, in this study, the virtual target-point-based processing technique, which is widely utilized in this research area, is employed to solve the local oscillation problem. This paper proposes a strategy for mitigating the oscillation in single-point obstacle path planning by using virtual target points.

The method of velocity potential field is employed to control the manipulator’s velocity directly in this paper. The extremely few time steps between velocities are implemented to attain smooth velocity interpolation. However, an oscillation path may generate a locally unsmooth trajectory, often along the edge of the repulsive potential field. Thus, a virtual target point method is utilized to guide the manipulator to quickly identify a location point outside the oscillation path and start a new path-planning process [30].

**Figure 5:**
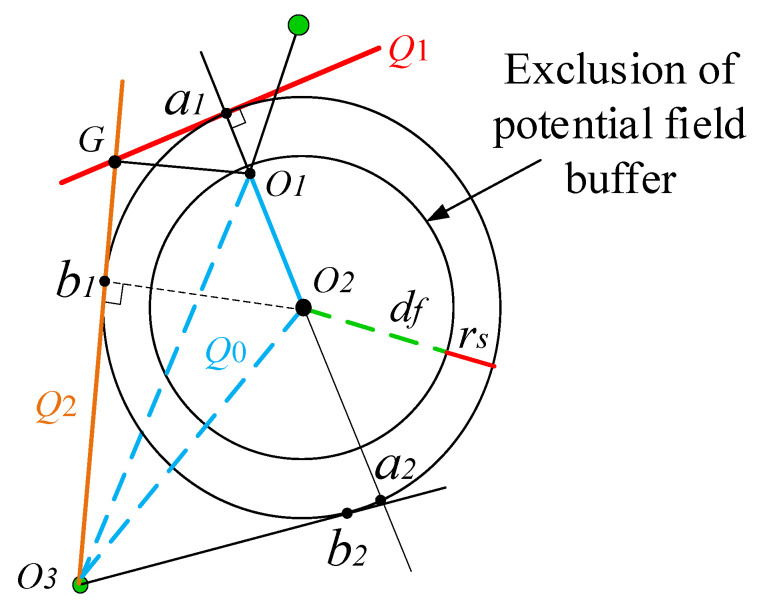
Diagram of the virtual target-point setting method.

To begin with, it is necessary to distinguish the oscillation section. In this study, the manipulator receives velocity data with an interval of 0.5 s to avoid any interference with real-time processing speed. To ensure efficient processing, a three-step over-planning method is employed, in which the manipulator calculates three sets of joint angles backward after calculating one set, and if the rejection speed appears repeatedly twice, an oscillation situation is confirmed. Once the manipulator confirms the onset of oscillation, a new virtual target point, G, is established to guide the manipulator out of the oscillation section. The positioning method of the virtual target point G is indicated in Figure 5.

In Figure 5, points $O_{1}-O_{3}$ denote the end coordinates of the manipulator upon entering the oscillation region, the center point coordinates of the obstacle and the target point coordinates, respectively. As the three points do not fall on the same straight line, they determine a plane, designated as $Q_{0}$. This plane serves as the tangent of the sphere in the repulsion potential field, as seen in Figure 5. Connecting the points $O_{1}O_{2}$ and the outer circle intersect at points $a_{1}$ and $a_{2}$. A tangential plane $Q_{1}$ is formed, which is perpendicular to the plane $Q_{0}$. Connect $O_{1}O_{2}$ and the outer circle to intersect at point $a_{1},a_{2}$, and make a tangent plane perpendicular to the plane of $Q_{0}$ over $a_{1}$, defined as $Q_{1}$. Make a tangent plane to the outer circle over point $O_{3}$, and define the tangent plane where $b_{1}$ is located as $Q_{2}$. The intersection point of $Q_{0}$, $Q_{1}$, and $Q_{2}$ is the virtual target point. The coordinates of the virtual target point G can be determined by correlating the three-plane equations. The manipulator moves to the virtual target point G from its initial position of oscillation. Once reaching the virtual target point, the manipulator moves straight towards the real target point. In this process, the path planning of the manipulator will be re-run. The virtual target point will be used as the starting point for re-planning, the original target point will be used as the termination point for planning, and the path planning will be performed using the improved VPF algorithm. The manipulator will continue to move towards the original target point.

## 3. Simulation and Experiments

The velocity potential field method-based obstacle avoidance algorithm for manipulator trajectories, presented in this study, can efficiently plan smooth motion paths that avoid collisions and mitigate potential local oscillations. To ascertain its effectiveness and real-time performance, experiments were performed using both simulation software and a laboratory environment. The data and graphical data offered in this work are derived from the simulations and the practical experiments undertaken by the researchers in this paper.

### 3.1. Simulation of Improved Velocity Potential Field Method and Comparative Analysis

#### 3.1.1. Obstacle-Free Simulation Verification

**Figure 6:**
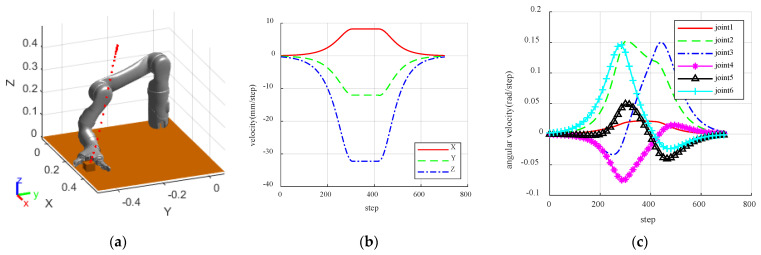
The results of path planning without obstacles. (**a**) The results of simulation; (**b**) The velocity in Cartesian; (**c**) The velocity in joint space.

Assuming that the maximum speed of the manipulator is within its speed limit, the trajectory of the manipulator from the initial position to the target position is shown in Figure 6a. Set the maximum attractive velocity boundary radius to $d_{max}=0.35$, the attractive velocity dividing factor to $d_{1}=0.15$, and the intensity coefficient of attraction speed to α=0.15, with a potential field-based step planning interval of 1 s. Set the range parameters of the repulsive potential field to $d_{f}=0.15$ and to $d_{s}=0.1$. Set the joint angles of the initial and target points of the robot arm as shown below:

(5) $$\left\{\begin{array}{l} q\_inti=\left[\begin{array}{cccccc} -1.6581 & -0.0873 & -1.7628 & 1.0996 & -1.8326 & -1.8326 \end{array}\right]\\ q\_end=\left[\begin{array}{cccccc} -1.3090 & 1.6232 & -0.9774 & 0.2967 & -1.6754 & -0.6807 \end{array}\right] \end{array}\right.$$

The obstacle is a sphere with a radius of 0.04 m with spatial location coordinates $obs=\left[\begin{array}{ccc} 0.4 & -0.3 & 0.3 \end{array}\right]$. To further examine the variations in joint velocity and arm end velocity in the absence of obstacles, the step planning time for the manipulator is modified to *t* = 0.01 s. Fine interpolation is conducted to obtain more precise results, and the velocity variation is shown in Figure 6b,c.

As observed in Figure 6b, cartesian space velocity, the end is guided by the attraction velocity, which is zero at the initial point. Then it advances to the maximum allowable speed with a smooth curve in a set period of time, and then falls smoothly to zero when approaching the target point. This relates to the functional definition of an “S-curve-like” function. Running below the speed barrier ensures the hardware safety of the manipulator throughout the operation. It can be seen from Figure 6c that during the whole operation of the manipulator, the speed of each joint is zero at the beginning and the end points, and that there is no impact. The overall joint speed is smooth and jitter-free, and the whole operation is smooth and matches the actual operation requirements. In the absence of obstacles, each joint of the manipulator generally exhibits smoothness, with no significant abrupt changes. This further confirms the viability of the designed attractive velocity function and affirms that the manipulator can complete a global path plan.

#### 3.1.2. Simulation of Local Oscillation

**Figure 7:**
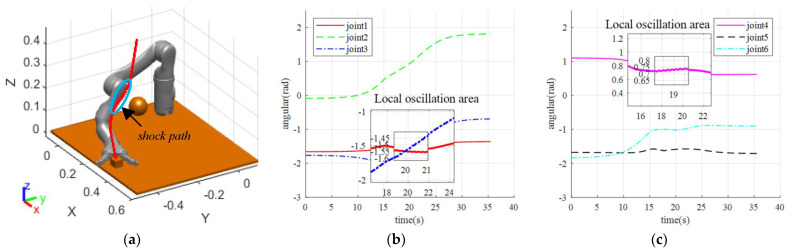
Simulation results when localized oscillations occur. (**a**) Obstacle-avoidance path for manipulators when localized oscillations occur; (**b**) The angle change of joints 1–3; (**c**) The angle change of joints 4–6.

In the absence of oscillation strategy processing, multiple obstacles are placed in the manipulator workspace until the manipulator experiences localized oscillations during motion. This can verify the ability of the robot to autonomously avoid obstacles and observe oscillations in its path when passing near obstacles. The rejection velocity function parameters a, b, and c are specified, and the step duration is set to *t* = 0.1 s. The simulation results are shown in Figure 7a.

The manipulator moves from the initial position towards the target position. When encountering an obstacle on the way, under the effect of the obstacle repulsion speed, the combined speed-controlled robot smoothly deviates to the left and reaches the target position, which verifies the correctness of the repulsive potential field function. Thus, the manipulator has the ability of autonomous obstacle-avoidance path planning. However, when the obstacle is close to the line connecting the manipulator and the target during the operation, as no oscillation processing strategy is adopted, the joint angle will cause local oscillation, as shown in Figure 7b,c.

**Figure 8:**
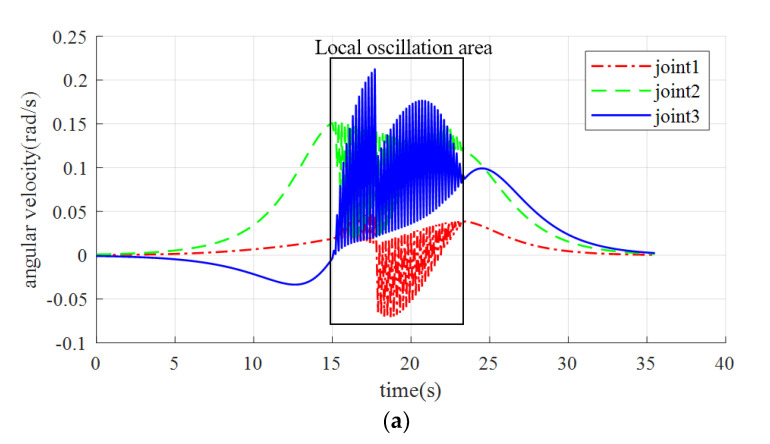

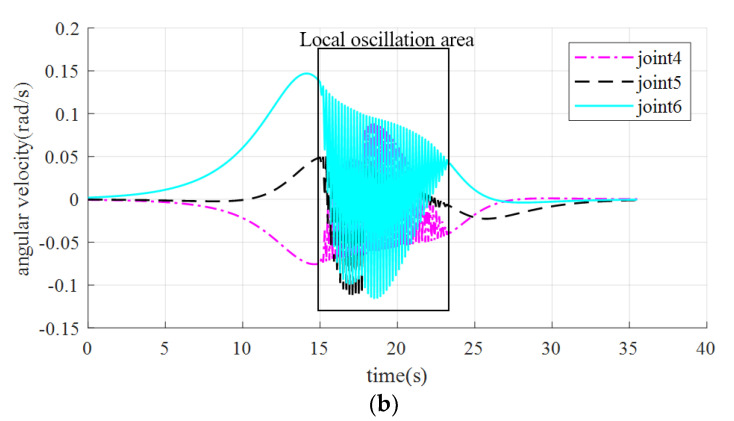
Joint velocity under local oscillation. (**a**) The speed variation of joint 1–3; (**b**) The speed variation of joint 4–6.

The oscillation segment was magnified, revealing that the manipulator is susceptible to trembling during operation due to the short planning duration of each step. Although the fluctuation range of the manipulator’s angle is small, typically around 0.01–0.02 rad, the arm experiences frequent angle jitter during this time frame. To further verify the effectiveness of the set repulsive potential field buffer zone and the speed fluctuation effect on the oscillation section, the speed simulation results for the scenario in Figure 8 were exported.

The velocity in the oscillation section displays apparent fluctuations in Figure 8, with joint velocities oscillating in the oscillation area, where the amplitude of oscillation increases and then decreases, corresponding to the manipulator traveling through the buffer zone, the repulsion zone, back through the buffer zone, and finally leaving the repulsion potential field. Therefore, the repulsive velocity potential field buffer is verified to be effective in the simulation, as the velocity amplitude does not change abruptly at the beginning or the end of the oscillation. However, because of the absence of a handling strategy for the oscillation situation described in the preceding section, the simulation was unable to avoid oscillation.

#### 3.1.3. Simulation after Setting Local Oscillation Processing Strategy

**Figure 9:**
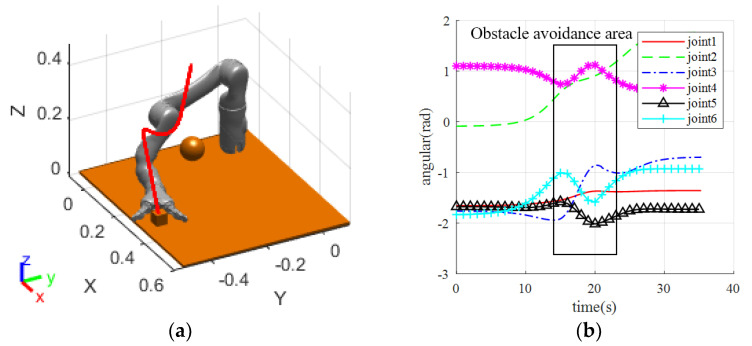

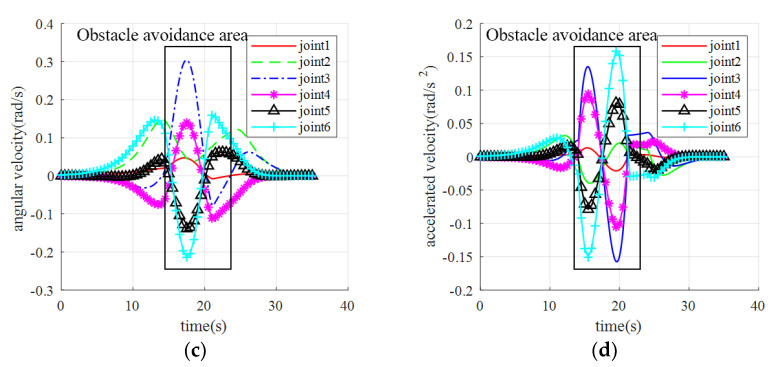
The results with the virtual target point improved. (**a**) The path of manipulator obstacle avoidance; (**b**) Joint angle of the manipulator; (**c**) Joint velocity of the manipulator; (**d**) Joint angular velocity of the manipulator.

Under the given conditions of the experiment, an additional radius “$r_{s}=0.05$ m“ beyond the buffer zone was set. With this setting, while keeping the obstacle positions unchanged, Figure 9 illustrates the results of the simulation, using Figure 7a as a reference. In Figure 9, for a more direct display, the step lengths are converted into time on the horizontal axis. After adopting the virtual target point strategy under local oscillation, the starting point of the oscillation-free path is searched, and then smoothed velocity planning is performed using a quintic polynomial trajectory-planning method to reach the target point smoothly after avoiding obstacles.

Compared to Figure 7b,c, in Figure 9b, the oscillation of each joint angle of the manipulator in the path section of the shock avoidance region disappears. Likewise, in Figure 9c,d, the velocity and angular velocity are smoother and more continuous, with only transient fluctuations observed at the initial and end positions of the shock avoidance path. This may be due to the approximation of acceleration at the beginning and end positions using the velocity-to-time ratio when using the five-times polynomial planning, leading to slight disturbances in the articulation. Although the obstacle-avoidance path generated by the new position point, which is obtained by increasing the safety radius, is usually inferior to the path generated by oscillation, this approach can result in smoother planning speed and similar operation step length since the number of set interpolation points is calculated based on the distance-to-velocity ratio, and it also avoids frequent tremors and shocks of the manipulator. From Figure 9c, it can be seen that the overall joint angular velocity of the manipulator is smooth and continuous during the operation, and there is no violent and sudden change of velocity when the oscillation occurs, so it can be determined that the method proposed in this paper can effectively eliminate the local oscillation phenomenon. Since the new position points are obtained under the condition of increasing the safety radius, the obstacle-avoidance paths generated by the new position points are not as excellent as the paths generated by the oscillations in most cases. But in this way, the planning speed can be smoothed, while avoiding frequent tremors and shocks of the manipulator.

### 3.2. Manipulator Real-Time Obstacle-Avoidance Planning Experiment

#### 3.2.1. Experiment Initialization Settings

**Figure 10:**
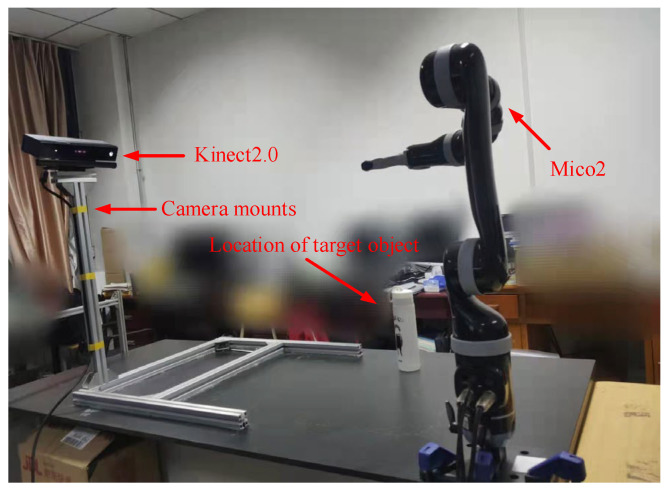
The experimental platform.

This paper’s experiments were conducted with a KINOVA Mico2 lightweight manipulator (Kinova Inc., Boisbriand QC, Canada) and a Kinect 2.0 vision sensor (Microsoft, Redmond, WA, USA). The primary hardware of the computer comprises an Intel i5-4590 CPU (Intel, Santa Clara, CA, USA) and an AMD HD-6500 graphics card (AMD, Santa Clara, CA, USA), and the system usesVS2015 software with OpenCV3.4.1, Kinect, and related libraries. We collect human body data and display the recognition of human body joint points, along with storing the data in C++. We simulate the manipulator using the Robotics System Toolbox in Matlab2020b, and the data is transferred to the manipulator for experiments. Figure 10 illustrates the experimental platform that was assembled in this paper.

#### 3.2.2. Collision-Free Scenario Path-Planning Experiments

**Figure 11:**
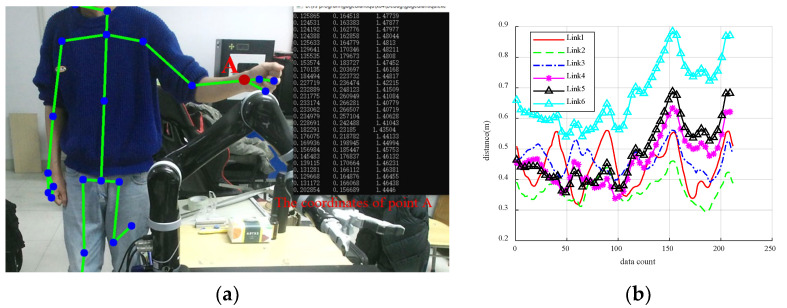
The real-time collected data. (**a**) The position of right-hand joint; (**b**) The distance between the manipulator and the obstacle.

The path-planning condition of the manipulator without the possibility of the collision was modeled and tested. The human right-hand was utilized as the experimental object, with the activity carried out inside a particular range outside the safety radius of the manipulator. The position of the right-hand joint point was obtained in real-time by a camera, and the distance between the right-hand joint point and each linkage of the manipulator envelope cylinder was determined by the control program. The real-time speed of the manipulator was also solved. As the human joint position was always ensured in the manipulator avoidance trigger position, which is beyond the range of the repulsive potential field, the simulation speed was delivered straight to the manipulator for the experiment. This was done to test the execution speed of the manipulator when no collision happens and to compare it with the simulation speed. During the operation of the manipulator, the information of the human right-hand joint is captured in real-time, as illustrated in Figure 11a, which depicts the left-hand due to the mirror image of the Kinect 2.0 camera. Figure 11b shows the real-time distances between the position of the right-hand joint and each linkage of the manipulator envelope cylinder.

In the process of human-machine collaboration, the safety distance between the manipulator and the human body is very important. Setting a reasonable safety distance can protect the safety of the operator very well. In order to ensure the safety of the human-machine collaboration experiment, in this paper, the safety distance between the manipulator and the human body is set to be 0.15 m. Figure 11b shows that the distance between the right-hand joint and the connecting rod remains above 0.15 m throughout the manipulator’s movement, preventing it from entering the range of the repulsive potential field. Therefore, the manipulator can move smoothly from its initial position to the target position without any possibility of collision with obstacles.

**Figure 12:**
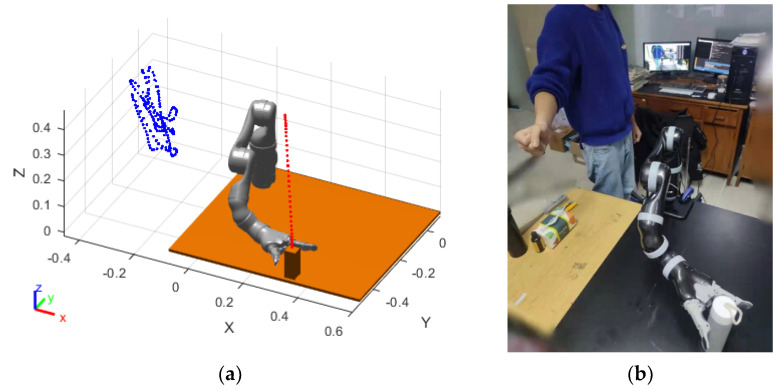
The path-planning result of the manipulator. (**a**) The result of MATLAB simulation; (**b**) The result of manipulator operation.

**Figure 13:**
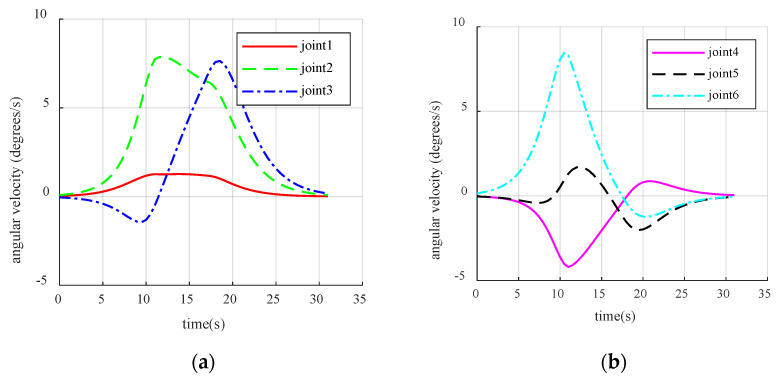

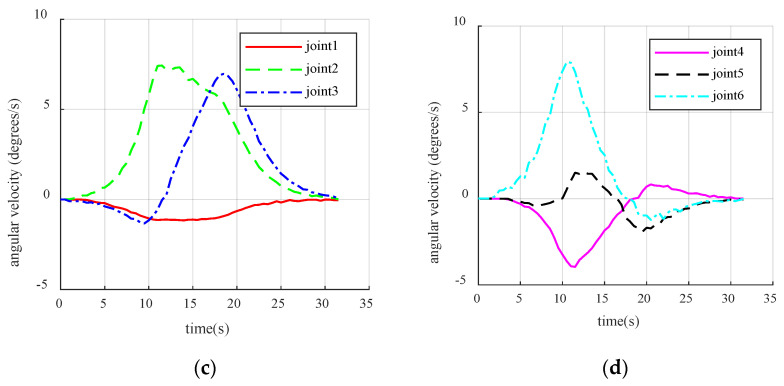
Comparison between simulation and experimental velocity of the manipulator. (**a**) Simulation velocity of manipulator joint 1–3; (**b**) Simulation velocity of manipulator joint 4–6; (**c**) Experimental velocity of manipulator joint 1–3; (**d**) Experimental velocity of manipulator joint 4–6.

In Figure 12a, the blue sample points in the simulation environment represent the movement trajectory of the human right-hand, which does not affect the movement of the manipulator, and allows it to reach the target position accurately. The range of the repulsive potential field is set to be 0.15 m in the simulation experiment. In the actual operation process, the distance between the manipulator and the arm joint has not reached the set repulsive distance. At the same time, the safety distance between the manipulator and the human body is set to be 0.15 m. Therefore, the distance between the manipulator and the arm never breaks through the safety distance during the running process. The whole experimental process is very safe, and the manipulator accurately reaches the target position. Figure 12b demonstrates that the final target pose of the manipulator in both the simulation and experimental environments is consistent. The speeds of the manipulator were collected at intervals of 0.5 s in both the simulation and experimental environments and are shown in Figure 13.

The two images in the upper part of Figure 13 represent the simulation results, while the two images in the lower part represent the experimental results. By comparing them, it can be observed that the speed of the manipulator during the experiment fluctuates slightly but is generally consistent with that in the simulation environment. Overall, although the speed exhibits some fluctuations, it remains around the set speed. Excluding the factors of the manipulator itself, the feasibility of speed control can be verified, and the accuracy of the manipulator to achieve the target posture by speed control can be ensured.

#### 3.2.3. Manipulator Real-Time Obstacle-Avoidance Experiment

**Figure 14:**
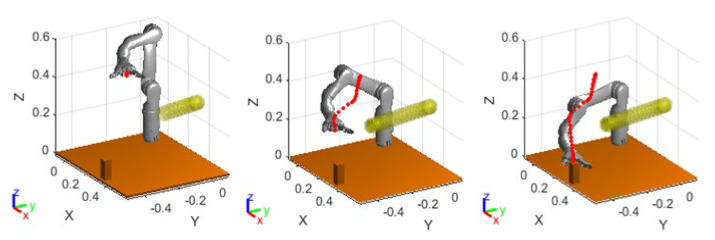
Real-time obstacle-avoidance planning of manipulator.

After verifying the feasibility of speed control of the manipulator in collision-free scenarios, this section verifies the real-time obstacle-avoidance path planning of the manipulator. The initial and target postures of the manipulator are set to be the same as in the previous section. Due to the complexity of real-time obstacle avoidance, and to avoid failures and other issues caused by calculation errors in the algorithm, a step-by-step verification method is adopted in this section for safety purposes. The human arm is set to move from the initial position [0.45, 0, 0.35] to the target position [0.3, −0.25, 0.25]. The simulated results of the manipulator motion process are shown in Figure 14.

**Figure 15:**
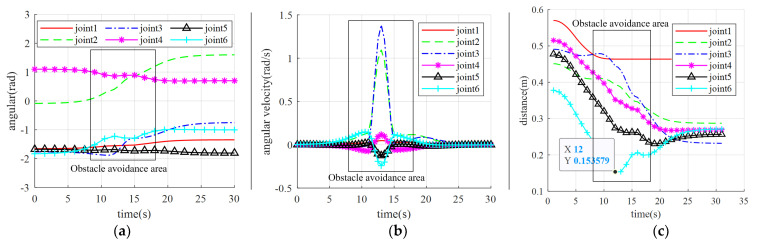
The results of the manipulator running. (**a**) Obstacle-avoidance angular of the manipulator; (**b**) Obstacle-avoidance angular velocity of the manipulator; (**c**) Distance between the manipulator and the obstacle in real-time obstacle avoidance.

As shown in Figure 14, the manipulator approaches the target in a straight line when avoiding obstacles correctly, then moves towards the virtual target point in the lateral and posterior direction after approaching the obstacle, and finally moves towards the target in a straight line after leaving the repulsive potential field, completing the entire path planning. The experiment shows that the virtual target point enables the manipulator to turn in a short time, avoiding the occurrence of frequent oscillations. However, due to the existence of lower-level control of the manipulator, the command speed sent directly is unstable, and the changes in joint angles and velocities collected at an interval of 0.5 s during the motion of the manipulator are shown in Figure 15a. The manipulator executed the obstacle-avoidance action in the time range from the 8th s to the 18th s. As can be seen from Figure 15a, the change of the angle values of the joints of the manipulator in the obstacle-avoidance region is reasonably smooth, and there is no strong jitter, which fits the dynamic obstacle-avoidance criteria of the manipulator.

Figure 15b shows that the velocity profile of the manipulator is not smooth enough in the obstacle-avoidance sequence. Due to the setting of the virtual target point, the 2-axis and 3-axis of the manipulator need to steer for a short time, so the velocity change in the relevant axes at the beginning and end nodes of obstacle avoidance is also larger. However, the operation of the manipulator is relatively more stable compared to the frequent oscillations. The distances between the manipulator links and the obstacles during the entire process are shown in Figure 15c.

As the position of link 1 is fixed, the distance between the hand and link 1 remains unchanged after the hand trajectory reaches the target position. According to the curve information in Figure 15c, it can be seen that during the motion process of the manipulator, the distance between the end link 6 of the manipulator and the hand does not enter the set safety threshold of 0.15 m. Obstacle-avoidance planning begins after predicting the oscillation by three steps, so the minimum distance is close to 0.15 m but does not breach it before obstacle-avoidance planning starts.

**Figure 16:**
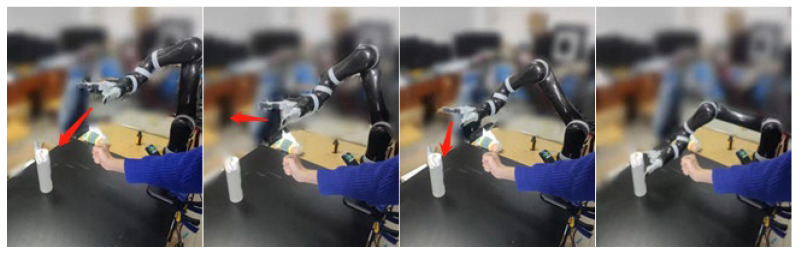
Real-time obstacle-avoidance experiment of manipulator.

After verifying the feasibility of obstacle avoidance in the simulation environment, experimental verification was conducted. According to the simulation results, the person’s right-hand was moved according to the above positions, and then the simulation speed was sent to the manipulator for path-planning experiments. During the experiment, it was found that when the manipulator moved at the simulation speed, the speeds before and after obstacle avoidance were faster than those in the simulation process. This discrepancy may be due to large changes in acceleration during obstacle avoidance and the effect of the manipulator’s speed, which is controlled at the lower level. Subsequently, this paper conducted experiments using angle control, and the operation of the manipulator was consistent with the simulation results. Finally, the manipulator avoided the obstacle and reached the target position, as shown in Figure 16.

**Figure 17:**
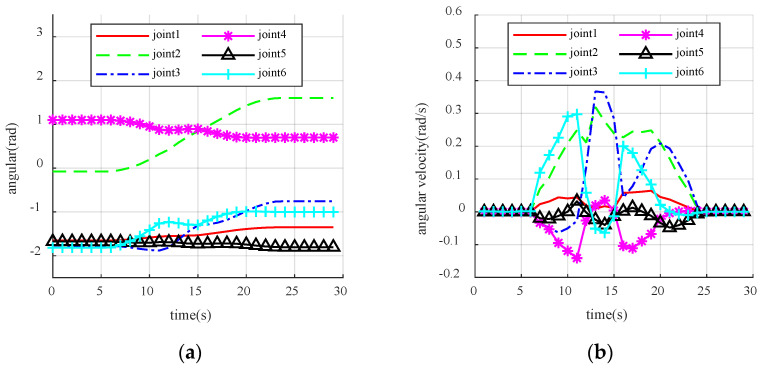
Obstacle avoidance and local oscillation of manipulator. (**a**) Obstacle avoidance of manipulator; (**b**) Obstacle-avoidance local oscillation of manipulator.

As shown in Figure 16, the manipulator gradually approaches the target in a straight line when correctly executing the obstacle avoidance, then moves towards the virtual target point in the lateral and posterior direction after approaching the obstacle, and finally moves towards the target in a straight line after leaving the repulsive potential field, completing the entire path planning. The experiment shows that the virtual target point enables the manipulator to turn in a short time, avoiding the occurrence of frequent oscillations. However, due to the existence of lower-level control of the manipulator, the command speed sent directly is unstable, and the changes in joint angles and velocities collected at an interval of 0.5 s during the motion of the manipulator are shown in Figure 17.

Table 1 compares the experimental results of the traditional VPF with the improved VPF proposed in this paper. From Table 1, it can be seen that the improved VPF proposed in this paper eliminates the local oscillation problem related to the traditional VPF algorithm and accomplishes the obstacle-avoidance task with a guaranteed safe distance. The safety performance in the process of human-machine collaboration is improved. Following experimental verification, the comparison of Figure 15a,b and Figure 17 shows that the joint angle of the running manipulator is consistent with the angle that was sent through simulation, and follows the correct path when running. However, due to the speed planning of the manipulator, the collected speed in the experiment differs from the simulation, mainly in axis 1 and axis 6. Nonetheless, the speed curve direction remains almost the same. The proposed obstacle-avoidance algorithm can control the speed and angle independently and measure the distance in real-time between the manipulator and the obstacle. When the manipulator enters the range of the repulsive potential field, it effectively reacts to obstacles, bypasses them, and smoothly reaches the target position.

## 4. Conclusions

This paper proposes a novel algorithm that uses the concept of velocity potential field for real-time obstacle-avoidance path planning of manipulators in three-dimensional space during human-machine collaboration. The proposed algorithm aims to ensure the optimal velocity continuity of the manipulator, minimize trajectory re-planning, and achieve simultaneous path and velocity planning. To address the sudden speed changes that occur at the beginning or end of the original two-dimensional algorithm, the “Slow-Fast-Slow” attraction velocity function is introduced to decrease the oscillation produced. Additionally, a virtual target point establishment method is presented to avoid local oscillations in the manipulator’s movement, followed by trajectory smoothing through the fifth polynomial. The feasibility of the algorithm was verified through the experiment’s real-time obstacle-avoidance path planning with a human upper limb placed near the manipulator. The manipulator completed the obstacle- avoidance experiment and reached the target position, demonstrating the algorithm’s effectiveness. However, due to the limited time and resources of the researchers, the obstacles were treated as single points in order to improve the computational efficiency of the algorithm. Future research is recommended to extend the parameterization of obstacles and optimize the strategy of the virtual target point setting method to achieve stable and smoother manipulator operation.

## Figures and Tables

**Table 1:** Comparison of experimental results between traditional VPF and improved VPF.

	Traditional VPF	Improved VPF
Arrived Goal	YES	YES
Local Collision	YES	NO
Breaking the Safe Distance	YES	NO

## Data Availability

Data sharing isn’t applicable to this article as no datasets were generated during the current study.
